# Comparative Performance of Linear Multielectrode Probes and Single-Tip Electrodes for Intracortical Microstimulation and Single-Neuron Recording in Macaque Monkey

**DOI:** 10.3389/fnsys.2017.00084

**Published:** 2017-11-15

**Authors:** Carolina G. Ferroni, Monica Maranesi, Alessandro Livi, Marco Lanzilotto, Luca Bonini

**Affiliations:** ^1^Dipartimento di Medicina e Chirurgia, Università degli Studi di Parma, Parma, Italy; ^2^Istituto Italiano di Tecnologia, Brain Center for Social and Motor Cognition, Parma, Italy

**Keywords:** silicon probes, electrical stimulation, macaque monkey, acute recording, chronic recording

## Abstract

Intracortical microstimulation (ICMS) is one of the most widely employed techniques for providing causal evidence of the relationship between neuronal activity and specific motor, perceptual, or even cognitive functions. In recent years, several new types of linear multielectrode silicon probes have been developed, allowing researchers to sample neuronal activity at different depths along the same cortical site simultaneously and with high spatial precision. Nevertheless, silicon multielectrode probes have been rarely employed for ICMS studies and, more importantly, it is unknown whether and to what extent they can be used for combined recording and stimulation experiments. Here, we addressed these issues during both acute and chronic conditions. First, we compared the behavioral outcomes of ICMS delivered to the hand region of a monkey's motor cortex with multielectrode silicon probes, commercially available multisite stainless-steel probes and single-tip glass-coated tungsten microelectrodes. The results for all three of the probes were reliable and similar. Furthermore, we tested the impact of long-train ICMS delivered through chronically implanted silicon probes at different time intervals, from 1 to 198 days after ICMS sessions, showing that although the number of recorded neurons decreased over time, in line with previous studies, ICMS did not alter silicon probes' recording capabilities. These findings indicate that in ICMS experiments, the performance of linear multielectrode silicon probes is comparable to that of both single-tip and multielectrode stainless-steel probes, suggesting that the silicon probes can be successfully used for combined recording and stimulation studies in chronic conditions.

## Introduction

The electrical stimulation of brain tissue marked the beginning of modern electrophysiology and strongly contributed to neuroscientific progress by allowing researchers to study brain organization and function in many animal species, including humans. Initially, electrical stimulation could be applied only to the cortical surface and induced gross activation that involved large neuronal populations (Penfield and Boldrey, 1937). Subsequently, the development of thin tip-electrodes, which could be inserted into the brain tissue (Asanuma and Sakata, 1967; Asanuma and Rosén, 1972), enabled the delivery of extremely localized intracortical microstimulation (ICMS) with high temporal precision. From the dawn of electrophysiology to the present, there has been tremendous progress in the terms of tools and techniques to artificially triggering neuronal activity, and neurotechnologies for ICMS are now being exploited not only for deepening our understanding of brain functioning (Cohen and Newsome, 2004; Graziano, 2016), but also for treating different brain diseases (Perlmutter and Mink, 2006; Schiff et al., 2007; Guridi and Alegre, 2016; Pais-Vieira et al., 2016), as well as for bidirectional neuroprosthetic applications (Lebedev and Nicolelis, 2006; Bensmaia and Miller, 2014; Flesher et al., 2016).

A crucial issue in ICMS applications consists in relating the electrically induced neural activation with the perceptual, cognitive, and/or behavioral outcomes, and this relationship has been quite extensively investigated using tip-electrodes (e.g., Salzman et al., 1992; Cooke and Graziano, 2004; Afraz et al., 2006; Lanzilotto et al., 2015a,b; Verhoef et al., 2015). However, in recent years, several new types of linear multielectrode probes have been developed. They have allowed researchers to record neuronal activity at different depths along the same cortical site simultaneously and with high spatial precision, to perform advanced functional studies of cortical laminae (Pettersen et al., 2006; Hansen et al., 2012; Glabska et al., 2016), and to artificially manipulate neural activity with ICMS (Lanzilotto et al., 2016). Nevertheless, the bulk of the evidence regarding the impact of ICMS on neural tissue derives from studies in which tip-electrodes were used. In particular, the volume of brain tissue involved by tip-electrode stimulation was deemed to increase with increasing current intensity. Stoney et al. (1968), for example, estimated that a spherical volume of 100–450 μm around the electrode tip was involved by stimulations with currents intensities ranging from 10 to 100 μA. However, a recent study that used calcium imaging to directly visualize the neurons activated by locally delivered electric pulses showed that the volume of tissue involved is mostly independent from the current intensity: indeed, with the increase of current intensity the number of neurons triggered by the stimulation increased, but within a similar volume of tissue. This finding is compatible with the idea that ICMS effects are based on a direct activation of axons in a volume of tens of microns in diameter (Histed et al., 2009). Even in this latter study, the current was delivered through an electrode tip, thus leaving unclear whether and to what extent the stimulation capability of probes with different geometries, such as linear multielectrode probes, is actually comparable to that of conventional tip electrodes.

In this paper, we compared the behavioral outcomes in terms of threshold of evoked movement or muscle twitches of ICMS delivered to the monkey's primary motor cortex with multielectrode silicon probes (Herwik et al., 2011), commercially available multisite stainless-steel probes and single-tip glass-coated tungsten microelectrodes. The silicon probes' stimulation capabilities were first compared with those of the other tested probes during acute experiments. Furthermore, to evaluate silicon probes' neural recording capabilities during combined recording and ICMS studies, we also tested the impact of long-train ICMS delivered through chronically implanted probes at different time intervals, from 1 to 198 days after ICMS sessions.

## Materials and methods

Experiments were carried out on one *Macaca mulatta* (male, 7 kg), previously used for other neurophysiological experiments (see below). Before recordings, the monkey was habituated to sitting in a primate chair and to interacting with the experimenters. It was then trained to perform the visuomotor tasks described in previous studies (Bonini et al., 2014b) using the hand contralateral to the hemisphere to be recorded. When the training was completed, a head-fixation system and a plastic recording chamber were implanted under general anesthesia over the right frontal cortex, as described in the sections below.

This study was carried out in accordance with the recommendation of the European law on the humane care and use of laboratory animals (directives 86/609/EEC, 2003/65/CE, and 2010/63/EU). All experimental protocols were approved by the Veterinarian Animal Care and Use Committee of the University of Parma (Prot. 78/12, 17/07/2012 and Prot. 78/91, 08/07/2015) and authorized by the Italian Ministry of Health (D.M. 294/2012-C, 11/12/2012, and Aut. 48/2016-PR, 20/01/2016).

### Sequence of experiments

We performed both acute experiments, in which a single electrode/probe was temporarily inserted into the brain and then retracted at the end of the experimental session, and chronic experiments, in which probes were permanently implanted in the brain tissue for several months.

After acute single-cell recording experiments with different types of linear multielectrode probes were conducted (Bonini et al., 2014a,b,c; Maranesi et al., 2014, 2015), tridimensional (3D) silicon probes were chronically implanted in the mesial pre-supplementary motor area F6 in order to perform single-neuron recordings and, at the end of the recordings, ICMS studies (Lanzilotto et al., 2016). Following this latter experiment, the chronically recorded activity was checked again the day after each stimulation session as well as up to 198 days after ICMS in a subset of the implanted probes (see Results section).

The comparative tests of ICMS with different electrodes/probes, the main focus of this study, were carried out in the hand motor region of the right hemisphere, where the recording chamber for acute experiments had been implanted. Hence, neither additional surgeries nor significant additional stress to the animal were implied. These data were collected in a total of nine independent sessions.

### Surgical protocols

All surgeries were performed under general anesthesia (ketamine hydrochloride, 5 mg/kg intramuscular [i.m.] and medetomidine hydrochloride, 0.1 mg/kg i.m., repeatedly administered during the surgery). Dexamethasone and prophylactic broad-spectrum antibiotics were administered pre- and postoperatively. Furthermore, analgesics were administered intra- and postoperatively. Dexamethasone administration was continued for 1 week after the surgery. During all surgeries, hydration of the monkey was maintained with continuous infusion of saline solution. A heating pad stabilized the monkey's body temperature throughout the surgical procedure. Heart rate, respiratory depth, and body temperature were continuously monitored. Upon recovery from anesthesia, the animal was returned to its home cage and closely monitored until complete recovery.

### Chronic neuronal recordings and ICMS procedures

The neuronal recordings described in this paper were performed by means of two 3D arrays of 64 sputter-deposited platinum electrodes arranged into 2 parallel lines of 4 shafts, each of which had eight recording channels. Probes were implanted with a vertical approach, approximately 1 mm laterally to the mesial wall. Previous reports provide more details on the methodology of probe fabrication (Herwik et al., 2011), assembly (Barz et al., 2014), and implantation (Bonini et al., 2014a; Lanzilotto et al., 2016).

The signal was amplified and sampled at 40 kHz with a 16-channel Omniplex recording system (Plexon, Dallas, TX), and recorded in sets of 16 channels. All final quantitative analyses were performed offline, as described in the subsequent sections. From the same chronically implanted probes, ICMS was performed at the end of the recording sessions. Monopolar, biphasic trains of cathodic square-wave pulses were delivered through a constant current stimulator (PlexStim, Plexon, Dallas, TX), with the following parameters, which are based on previous ICMS studies on area F6 (Luppino et al., 1991): total train duration 500 ms, single-pulse width 0.2 ms, pulse frequency 300 Hz. The current intensity ranged from 1 to 100 μA and was controlled on an oscilloscope by measuring the voltage drop across a 10 KΩ resistor in series with the stimulating electrode. The stimulator had an automatic electrode discharge feature that removed, during the inter-pulse interval and any time the electrode was not stimulating, charge previously deposited. More details on the ICMS experiment have been provided elsewhere (Lanzilotto et al., 2016). Movements were considered to be evoked by ICMS when two experimenters, observing the animal during pulse delivery, independently and repeatedly identified the same joint displacement or muscular twitch. The lowest current intensity capable of evoking movements in 50% plus one of the stimulations was considered the threshold.

The same procedure was employed to compare the effects obtained during acute ICMS experiments targeting the hand motor region with different types of electrodes/probes. In this case, however, the stimulation-train duration was set to 50 ms and the initial current intensity to 40 μA, in line with the higher electrical excitability of the primary motor cortex (Kwan et al., 1978; Sessle and Wiesendanger, 1982; Maranesi et al., 2012). When a clear ICMS-evoked response was observed, the current intensity was first lowered in steps of 10 μA, and then in smaller steps (up to 1 μA), in order to precisely identify the stimulation threshold of the cortical site. The comparative ICMS experiment was carried out by using (i) a 16-channel linear silicon probe (Herwik et al., 2011; Bonini et al., 2014a, distributed by ATLAS Neuroengineering, Belgium, length 8 mm, rectangular section 100 μm, single sputter-deposited platinum electrode diameter 35 μm, inter-electrode pitch 250 μm), (ii) a 16-channel linear stainless-steel multielectrode probe (U-Probe, Plexon, circular section diameter 185 μm, single platinum/iridium electrode diameter 15 μm, inter-electrode pitch 250 μm), and (iii) a glass-coated tungsten single-tip electrode (Alphaomega Engineering, circular section diameter 250 μm). The impedance of all electrodes (single-tip electrodes and recording sites of the linear multielectrode probes) was measured (at 1 kHz) with the electrode in the brain, before and after each acute ICMS session, and ranged from 0.4 to 1.3 MΩ. We performed nine sessions with each electrode/probe, as follows. The single-tip electrode was inserted at a depth of 5 mm from the surface of the intact dura. ICMS was delivered to identify the current threshold with the methodology described above. The electrode was then retracted in steps of 500 μm, and ICMS was repeated in the same way in all steps to identify the thresholds, until the end of the cortex was reached (typically, about eight electrically excitable sites were found). Similarly, the laminar probes were inserted at a depth of 5 mm from the surface of the intact dura, and ICMS was then delivered from alternated sites, hence every 500 μm, to identify the current threshold in each of them. To more directly compare the stimulation properties of the three probes, in each session they were inserted in the same cortical site, varying the order of probes insertion in each session to avoid any bias due to accumulating cortical damage following repeated penetrations. This was achieved by identifying the site visually under a surgical microscope and trying to insert each probe in the hole and along the track left by the previously inserted one. The penetration angle was approximately perpendicular to the cortical surface. Single-tip electrodes and probes were inserted with a manually driven stereotaxic micromanipulator mounted on the recording chamber. Single-tip electrodes and the U-Probe were inserted through a guide-tube placed in contact with the dural surface (after the tip of the electrode/probe had been placed in the correct position), while silicon probes were inserted by means of a vacuum holder and then left floating during the stimulation procedures (see Bonini et al., 2014a for more details).

### Data analyses

Single-unit activity (SUA) was detected by applying to the filtered (300–6,000 Hz) wide-band activity a negative threshold corresponding to 2.5 standard deviations from the mean peak height. Spikes were then sorted using standard principal component and template matching techniques, provided by dedicated offline sorting software (Plexon Inc.), as described elsewhere (see Bonini et al., 2014a).

## Results

### ICMS of the motor cortex during acute experiments

**Figure 1:**
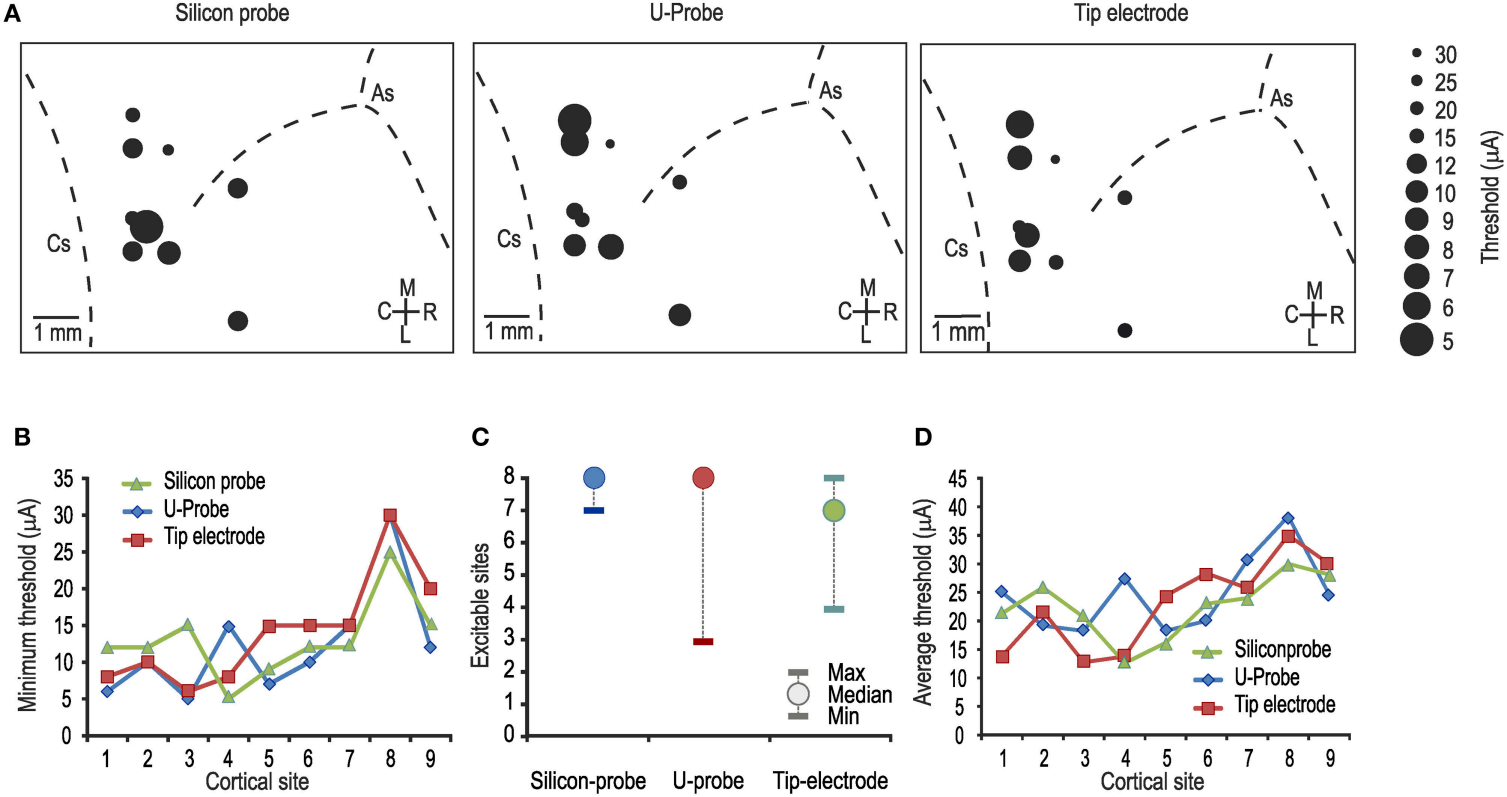
**(A)** Map of the penetrations carried out with each of the tested probes. In each map, the diameter of the dots indicates the minimum current-intensity threshold obtained in each cortical penetration, as specified in the legend on the right. As, arcuate sulcus; Cs, central sulcus. **(B)** Comparison between the minimum thresholds obtained with the three probes in the same cortical sites [1-way ANOVA, *F*_(2, 24)_ = 0.17]. Number from 1 to 9 represent cortical sites investigated one after the other in temporal order. **(C)** Median and range (minimum and maximum) of the number of electrically excitable cortical sites over the total number of stimulated sites per penetration (*n* = 8). **(D)** Comparison between the average thresholds obtained with the three probes in the same cortical sites [1-way ANOVA, *F*_(2, 24)_ = 0.27].

We performed nine acute penetrations with each of the three probes in a restricted cortical region that included the crown and adjacent convexity of the right central sulcus (the transitional region between the primary motor and premotor cortex; see Figure 1A). We define “cortical site” as each position in which a penetration was carried out with all three of the probes under comparison (see Materials and Methods). In general, we explored the hand representation in a region encompassing the motor/premotor cortical areas: indeed, the most frequently evoked behavioral reaction following ICMS consisted in fast twitches of the fingers, typically involving flexion of all the fingers toward the hand palm, and twitches of a single finger, usually the thumb and/or the index finger, at the lowest current intensities.

**Figure 2:**
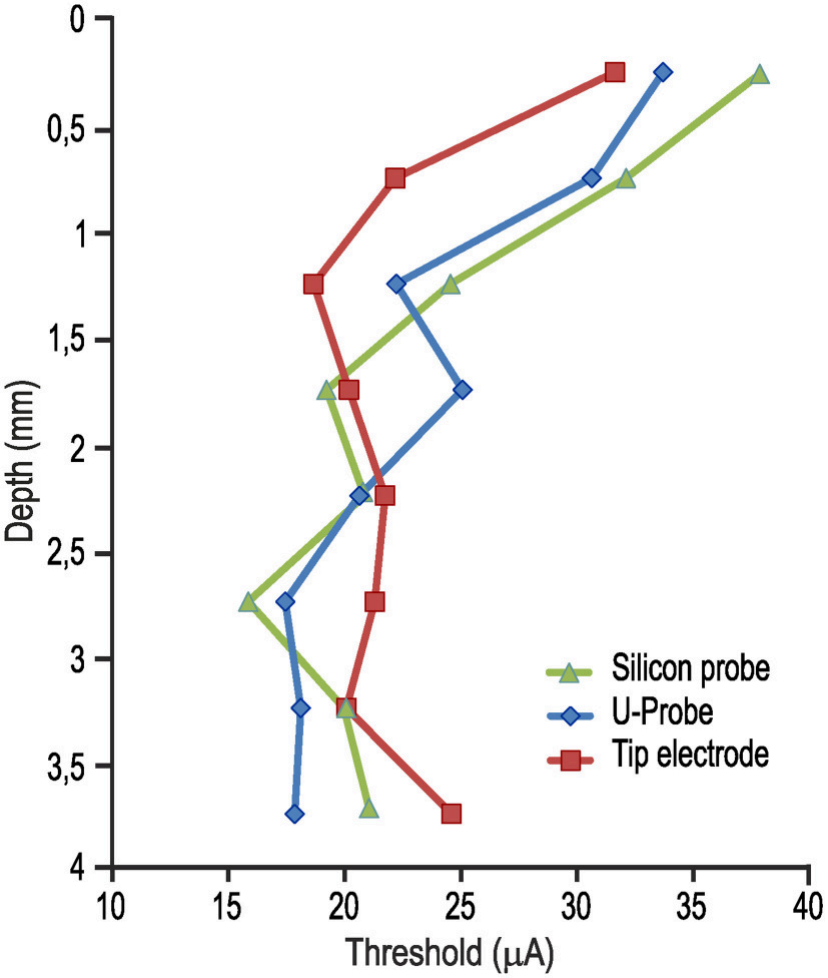
Average current-intensity threshold obtained with ICMS delivered through each of the three probes as a function of the cortical depth of the stimulated sites [1-way ANOVA, *F*_(2, 21)_ = 0.10].

Figure 1A shows the lowest current intensity threshold obtained with each probe at each cortical penetration. The formal comparison of the lowest thresholds obtained with the three types of probe for each cortical site indicates no significant differences (Figure 1B). The overall electrical excitability of each cortical penetration was also compared among probes both in terms of the number of electrically excitable sites relative to the total number of stimulated sites (Figure 1C) and in terms of the average thresholds among all the investigated sites for each penetration (Figure 1D). Again, no significant differences were observed. Furthermore, we explored the possible relationship of local thresholds obtained at different cortical depths (i.e., from superficial to deeper layers) with each probe: we did not find any significant differences among probes (Figure 2), and for all of them the typical increase in electrical excitability (lower current-intensity thresholds) of the deepest cortical sites (likely corresponding to layers III/V), relative to the more superficial sites, was evident. All together, these findings indicate that different local anatomo-functional specificities of cortical sites (i.e., distance from the central sulcus or from layer V), which are typically associated with differences in electrical excitability, appear to be similarly captured by all three probes, which, in turn, did not differ significantly from each other in terms of stimulation capabilities.

**Figure 3:**
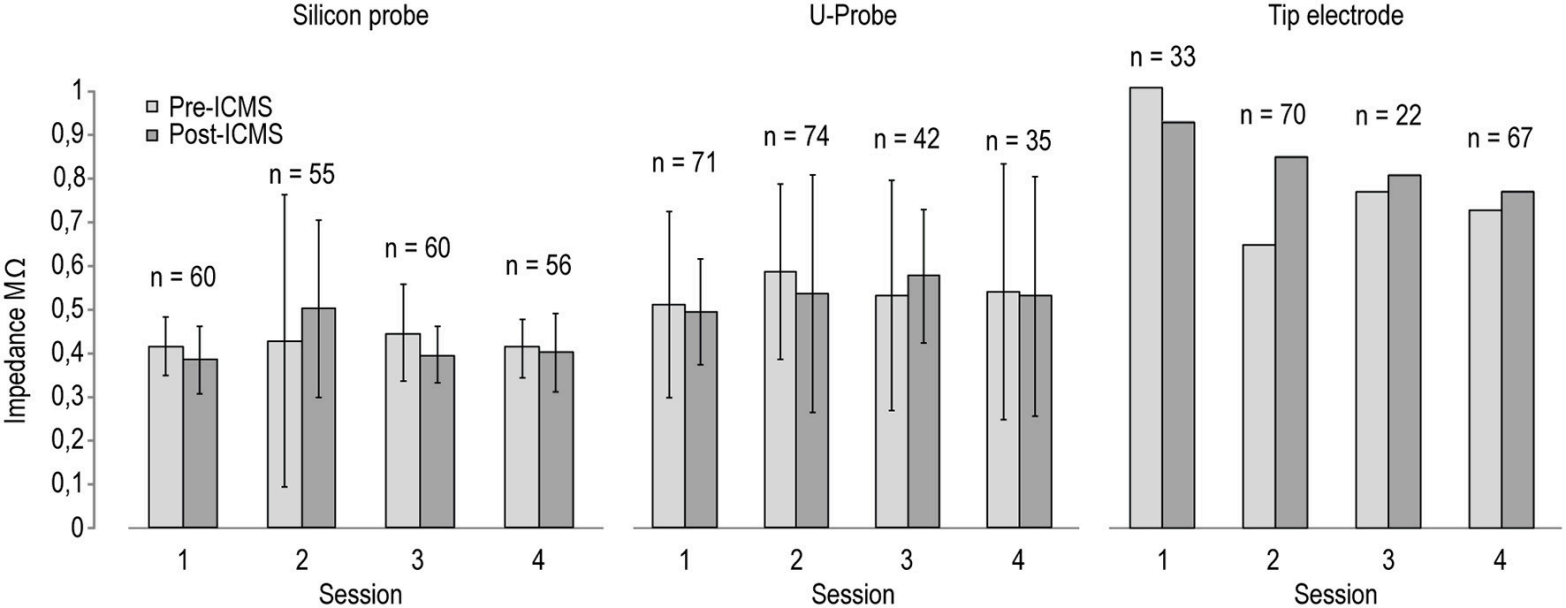
Impedance of the stimulating electrodes (*n* = 8 for silicon probes and U-Probes, *n* = 1 for the tip electrode) measured before (black) and after (gray) four different ICMS sessions. The number on the top of each pair of histograms indicates the number of ICMS trains delivered between the two measurements in each session.

Figure 3 shows the measurements of electrodes' impedances at various time intervals before and after ICMS sessions. In spite of some variation following ICMS, electrodes' impedances remained generally stable in all three probe types, suggesting the feasibility of recording and stimulation experimental protocols. Because we used silicon probes to conduct chronic recording and stimulation experiments that have been described in recent studies (see Lanzilotto et al., 2016; Barz et al., 2017), we were able to test the recording performance of chronically implanted silicon probes before and after ICMS sessions, as described in the following section.

### ICMS and neuronal recording from the mesial frontal cortex in chronic conditions

In recent studies, we have described the functional properties (Lanzilotto et al., 2016) and the stability of SUA chronically recorded over a period of 42 days from two 64-channel 3D probes (Barz et al., 2017). In this study, we focused on a set of 32 channels of one of the two 3D implanted probes (4 silicon shafts with 8 channels each one). In this set of channels, we monitored the activity before ICMS sessions (carried out on day 42) as well as after them, either on the same day or at different subsequent time intervals (44 and 198 days after ICMS).

**Figure 4:**
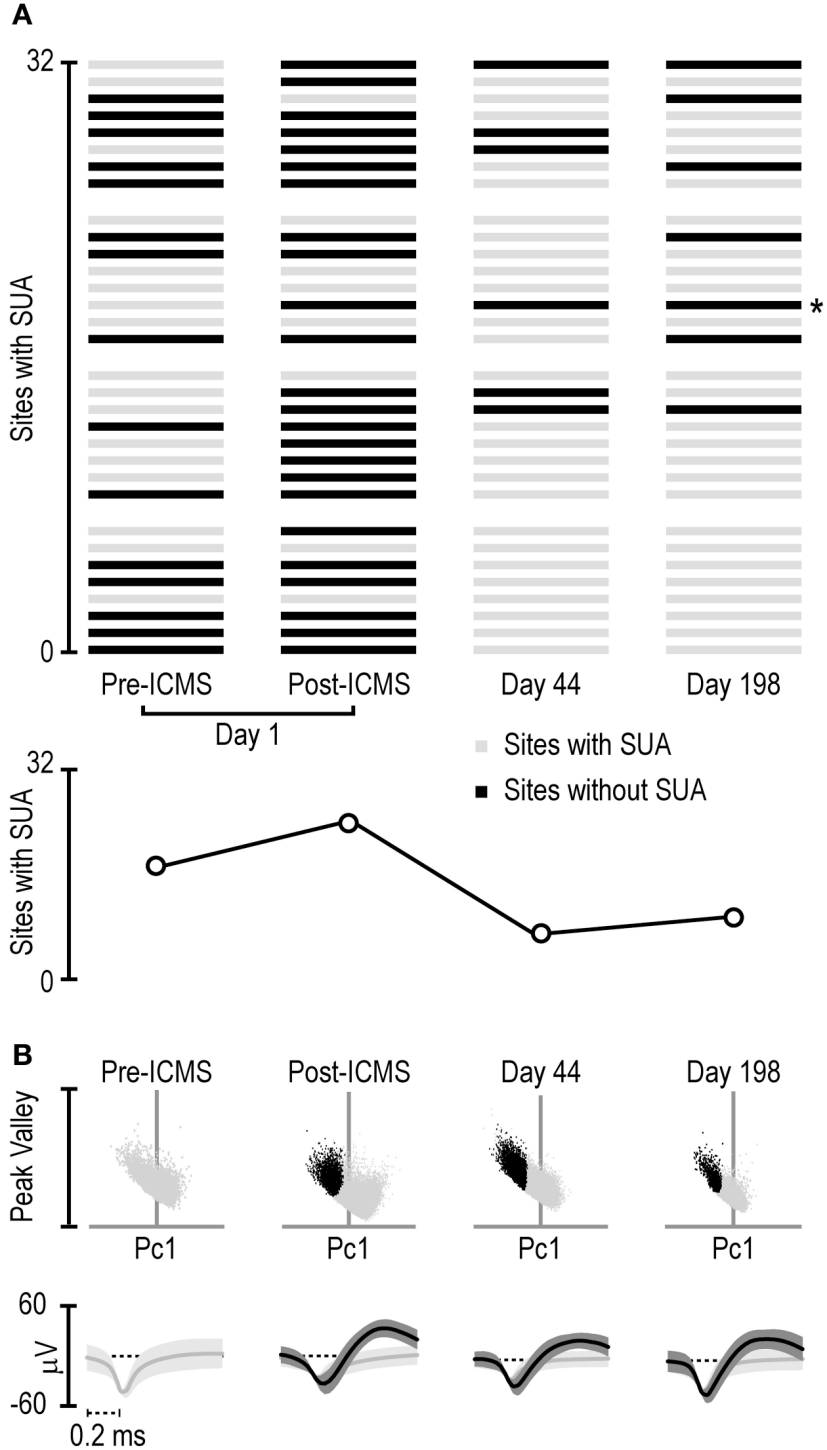
**(A)** Distribution of single-unit activity detected with the 32 channels of the 4-shaft silicon probe monitored four times during a period of 198 days subsequent to the ICMS session (day 1 pre- and post-ICMS, day 44 and day 198). Each set of 8 channels represents 1 shaft, and the distribution of the channels is ordered on the basis of cortical depth (deepest sites on the bottom). The asterisk on the right of the panel indicates the channel whose activity is shown in **(B)**. **(B)** Projection in a 2D space of all the waveforms exceeding a 2.5-standard-deviations threshold from the signal-to-noise of the band-pass filtered signal recorded from the channel labeled with the asterisk in **(A)** at different times. The 2D space is defined by the dimensions used to sort spikes into isolated single units, namely, the first principal component (horizontal axis) and the highest peak-valley waveform amplitude (vertical axis). Black dots represent waveforms attributed to a single unit relative to unsorted waveforms (light gray dots). Under each scatterplot, the average (± 1 Std Dev) waveform of the sorted single neuron (black) and remaining unsorted (gray) waveforms is presented.

Figure 4A shows that the absolute number of sites with clear-cut SUA before ICMS (15/32) not only did not decrease but even increased immediately after the stimulation (24/32). This effect is clearly due to the greater number of sites that began to exhibit SUA only after ICMS (*n* = 10) relative to those that exhibited the opposite effect (*n* = 1). Notably, most of the sites that initially showed SUA still did that after ICMS as well (14/16). Figure 4 also evidenced that after longer time intervals (44 and 198 days post-ICMS, that is, 86 and 240 days post-implantation) single units could still be isolated from some of the channels, in spite of an overall considerable reduction in the number of channels with SUA.

Figure 4B shows an example of the waveforms exceeding 2.5 standard deviations from the signal-to-noise threshold of the band-pass filtered signal recorded at one of the recording sites (marked with asterisk in Figure 4A) at each of the four time points. Whereas, no SUA could be detected before ICMS, a well-isolated single unit could be clearly detected from the signal recorded after ICMS on the same day. Furthermore, the same channel showed well-isolated SUA in the recording sessions carried out at both 44 and 198 days after the ICMS session (i.e., 86 and 240 days post-implantation). Although the example in Figure 4B may suggest a considerable stability even of the same single unit over a long period of time (198 days), it should be noted that similar wave-forms does not necessarily imply that they have been generated by the same neuron: indeed, we observed most often considerable changes in wave-form features over time. Stable wave-form features and functional properties in specific behavioral tasks over several weeks (which provide convergent evidence of single-cell recording stability) can be only occasionally observed (Barz et al., 2017).

## Discussion

In this study, we compared the performance of linear multielectrode silicon probes in acute ICMS experiments in the motor cortex of a monkey with the performance of linear multielectrode stainless-steel probes and classical tip electrodes. In addition, we verified the long-term single-neuron recording capabilities of chronically implanted silicon probes previously used for both ICMS and single-neuron recordings.

The results of the acute ICMS experiments showed that all three probes could trigger similar behavioral reactions at comparable current-intensity thresholds. We used a single tip-electrode for comparison because most extant ICMS studies on both motor and nonmotor brain regions have been carried out with this type of electrode. However, because we sequentially replicated cortical penetrations in the same position with different probes (in the same session and randomizing the sequential order across sessions), it could be argued that we failed to capture subtle differences among probes, thus producing false-negative results. Nevertheless, it is worth noting that, with all probes, the minimum and average current-intensity thresholds reflect the well-established properties of the motor cortex, that is, greater excitability of the cortical sites located closer to or within the anterior bank of the central sulcus (Maranesi et al., 2012) and in the deepest (likely layer V), as opposed to the superficial, cortical layers (Asanuma and Rosén, 1972). These findings are also in line with the relatively stable impedances measured before and after ICMS sessions on different days, which suggests that the tested probes constitute equally suitable options for ICMS studies despite the differences in electrode size and material (Cogan, 2008). The possibility of using silicon probes in highly stable, floating conditions (Bonini et al., 2014a) may render them particularly suitable for use in combined recording-stimulation experiments.

Of the tested probes, silicon probes have been also designed and assembled for chronic neural recording applications by several groups. Thus, the results of the acute experiments support the possibility of exploiting silicon probes for combined ICMS-recording experiments in chronic conditions as well. Indeed, in a recent study, we chronically implanted linear silicon probes in the monkey pre-supplementary motor area F6 to record single-neuron activity and, at the end of the recording sessions (42 days post-implantation), to deliver ICMS at all the available sites (see Lanzilotto et al., 2016). In another paper we presented data concerning silicon probe fabrication and assembly, as well as their functional validation during the first 42-day period after probe implantation (Barz et al., 2017). Here, we analyzed the data gathered from a subset of these chronically implanted probes in order to assess their single-neuron recording capabilities before and after ICMS as well as at various time intervals subsequent to ICMS, during a period of additional 198 days. The results showed that the yield of single units slightly increased, in the same day, from 0.5 single units/channel before ICMS to 0.75 units/channel after it, indicating that ICMS did not compromise the silicon probes' recording capabilities, which is in line with previous evidence (Rajan et al., 2015). The overall increased yield of single units following ICMS observed in the present study can be explained by the effect of the stimulation pulse on the interface between the recording sites and the non-neuronal surrounding tissue (Johnson et al., 2004, 2005): indeed, these latter studies suggested that the application of a single voltage bias to chronically implanted probes can constitute a viable way to transiently “rejuvenate” (for up to 1 week) chronically implanted probes.

Altogether, the present findings support the feasibility of novel approaches in which 3D arrays of silicon probes can be used for chronic recording and stimulation of neuronal activity from homogeneous volumes of cortical tissue, thanks to the uniform distribution of the recording sites along the shafts. Similar probes have also been used for chronic recordings in freely behaving animals (Michon et al., 2016) and interfaced with telemetric devices (Fan et al., 2011), which may even allow researchers to investigate high-order sensorimotor and sociocognitive functions in freely behaving large animals, such as nonhuman primates. Although we did not monitor the stability over time of ICMS-evoked behavioral effects, it has been shown in previous studies that they remain stable over periods of several months with chronically implanted electrode arrays (Callier et al., 2015).

An important limitation of the present study consists in the low number of observations carried out on a single animal with a single set of probes. A comprehensive retrospective report on a large set (*n* = 78) of microelectrode arrays chronically implanted in 27 monkeys over a period of about 15 years showed that the recording duration ranged from 0 to 2,104 days, with a considerable case-by-case variability and with a number of possible different failure modes (Barrese et al., 2013). Thus, although the present findings appear promising because of the different experimental possibilities they open up, they should be considered preliminary and will have to be integrated by additional and more extensively quantified future observations in order to better assess the cost-benefit ratio associated with their use.

## Author contributions

CF performed the experiments, analyzed the data, prepared figures and wrote and revised the manuscript; MM performed the experiments, analyzed the data and revised the manuscript; AL performed the experiments, analyzed the data and revised the manuscript; ML performed the experiments, analyzed the data and revised the manuscript; LB designed the research, supervised the experiments, wrote and revised the manuscript.

### Conflict of interest statement

The authors declare that the research was conducted in the absence of any commercial or financial relationships that could be construed as a potential conflict of interest.
